# Supplementary material for: Population interconnectivity over the past 120,000 years explains distribution and diversity of Central African hunter-gatherers
Source: Proc Natl Acad Sci U S A. 2022 May 17;119(21):e2113936119. doi: 10.1073/pnas.2113936119 (PMC9173804; doi:10.1073/pnas.2113936119)
Supplement: Supplementary File [file pnas.2113936119.sapp.pdf]

PNAS
www.pnas.org

# Supplementary Information for
# Population inter-connectivity over the past 120,000 years explains distribution and diversity of Central African hunter-gatherers

Cecilia Padilla-Iglesias*, Lane Atmore, Jesús Olivero, Karen Lupo, Andrea Manica, Epifanía Arango Isaza, Lucio Vinicius, and Andrea Bamberg Migliano

*Corresponding author.
Email: cecilia.padillaiglesias@uzh.ch

**This PDF file includes:**

Supplementary Text
Figs. S1 to S19
Tables S1 to S10
Legend for Movie S1
Legend for Datasets S1 to S4

**Other supplementary materials for this manuscript include the following:**

Movie S1
Datasets S1 to S4

## Supplementary Information Text

### Environmental Niche Modelling of distribution of CAHG using Favourability function

Besides their simplicity, GLMs are extremely powerful for modelling the distribution of species (or in our case populations) as a function of environmental conditions and have been previously shown to be useful in this particular study setting (1). They also allow us to calculate the favourability function (2-3), which is based on the output of GLMs but cancels out uneven proportions of presences and absences in the modelled data. It thus assesses the extent to which the environmental conditions change the probability of occurrence of an organism with respect to its overall prevalence in the study area. This is necessary because when the proportions of presences and absences are not equal within the sample, the logistic regression output within the function's domain is not symmetrical, but rather deviates towards the extreme that has a greater number of cases (4).

In order to obtain predicted favourability values across our study area, we obtained the probability of presence (P) across the region using a forward-backward stepwise logistic regression. Subsequently, the favourability value (F) for each square was calculated applying the function described by Real et al.(4):

$$F=[P/(1-P)]/[(n_1/n_2) + (P/[1-P])]$$

where $n_1$ and $n_0$ are the numbers of presences and absences, respectively. This function yields values ranging from 0 to 1, which are levelled in relation to the prevalence of the species and hence are different from probability or suitability values (5). For the extrapolation of F values, the following equation was used (4):

$$\boldsymbol{F} = \frac{\boldsymbol{e^y}}{\left(\frac{\boldsymbol{n_1}}{\boldsymbol{n_0}} + \boldsymbol{e^y}\right)}$$

where *y* is the logit link of the logistic regression equation, and e is the base of Napierian logarithms.

Predicted favourability values were converted to presence/absence predictions using a threshold value of F=0.5, which has been shown to be biologically meaningful, whilst also approximating the value that maximises sensitivity and specificity **(4)**(Table S10).

### Estimation of camp and CAHG population density in the present and past using Favourability

Following other studies that have found a positive relationship between population density and environmental favourability (1,6), we also examined the association between hunter-gatherer population density and environmental favourability in the 50 grid cells (N = 75 camps) for which camp-size data were available.

After calculating population densities at each of the grid cell, removing outliers, and confirming the expected wedge-shaped relationship (see Methods, Main text, Fig.S4), we fitted linear quantile regressions to the 50$^{th}$, 55$^{th}$, 60$^{th}$, 65$^{th}$, 70$^{th}$, 75$^{th}$, 80$^{th}$, 90$^{th}$, 95$^{th}$ and 99$^{th}$ percentiles, and the R1 measure (weighted sum of absolute residuals) was calculated in each percentile as a local measure of goodness-of-fit **(7)**.

Then, we estimated metapopulation sizes by first dividing the range of environmental favourability into three distinct categories, unfavourable: <0.2, medium: 0.2–0.5, favourable: >0.5. We then calculated the average CAHG population size empirically observed in grid cells for which

population sizes were available (after removing outliers). Using these figures, we then calculated the potential population size (PPS) for every grid cell in the study area, according to their favourability values. Finally, we summed all PPS values for the entire study area, but applied the following correction to take territoriality into account:

$$\text{Metapopulation} = \text{GPPS} \times \text{GCS} / \text{ASA}$$

where the metapopulation is the net potential population size; GPPS is the gross potential population size resulting from the sum of the PPS values; GCS is the size of a grid cell (i.e. 123 km2); and ASA is the average subsistence area estimated for CAHG (i.e. 1,079 km2)(Fig.S19).

**Estimation of past distributions of CAHGs from Favourability GLMs and validation with archaeological assemblages**

As for suitability, low values of favourability indicate that conditions are environmentally unsuitable for the presence of hunter-gatherer camps, whereas high values indicate that conditions are suitable. Hence, we also projected our Favourability GLM into each of the 1000- or 2000-year time slice from the present to 120,000BP to obtain probability maps in our area of interest for each time period.

However, it is important to note that it is more theoretically justified to extrapolate suitability values than favourability values to the past as the purpose of such extrapolation is to characterise past environments according to how suitable they are for hosting hunter-gatherer populations (i.e. how likely they are to have presences) rather than designing a discriminatory algorithm to discern where these population would or would have not been at specific points in time.

We then determined whether our estimated probability landscapes in the past matched areas actually occupied by hunter-gatherer populations in the same way as for our favourability model but using the threshold of F=0.5 to binarize favourability values at the cells containing archaeological assemblages, and therefore to determine the number of archaeological sites located in cells with predicted presences.

**Assessment of the relationship between ecology and rural population density**

Any observed relationship between bioclimatic or ecological variables and CAHG presence and/or density could be the product of environmental contraints acting specifically on hunter-gatherers populations or instead of more general constraints on human adaptability that apply to all populations regardless of their subsistence style.

To contrast these possibilities, we ran a general additive model to determine the relationship between rural population density and the predictors used in our ENM models to predict the presence and density of Central African Hunter Gatherers. We chose K=4 knots to prevent overfitting **(8)** and applied smoothing splines to all continuous parameters and included “Biome” as parametric term.

**Additional datasets of $^{14}$C dates**

To assess the robusticity of our results concerning the ability of our ENMs to predict the location and date of archaeological sites to particular choices regarding the inclusion criteria of particular $^{14}$C dates, we created two additional datasets.

First, although the $^{14}$C dates included from the sites located at Gombe Point and Rivière Denis are generally regarded as reliable and have been included in recent published surveys of Late Stone Age material in central Africa (9-11), some authors in the past expressed concerns due to them coming from bulk samples and potentially having been subjected to vertical disturbances

**(12)**. We therefore created an additional dataset excluding all dates from Gombe Point as well as Rivière Denis from the final dataset described in the Main Text (N=159).

Although worldwide the fabrication and use of ceramic pots by hunter-gatherers is both widespread and ancient (see for example Budja et al. **(13)**, Lupo et al. **(14)**, Clist,**(15)**; Jordan et al.**(16)** some authors interpret pottery use as evidence of storage and therefore deviations of a “pure” hunting and gathering subsistence economy **(17)**. Hence, we made a dataset comprising the subset of sites from the final dataset described in the Main Text that did not show any evidence of pottery use (N=145).

We then applied the same filtering procedures as described in the main text to these two additional datasets to aggregate multiple dates from each grid cell and 1000- or 2000-year time interval.

**Private allelic diversity within and across CAHG groups**

To determine the extent of gene-flow between hunter-gatherer groups across Central Africa, we also calculated, for each population its allelic richness (number of distinct alleles in the population), private allelic richness (number of alleles private to the population) and the mean number of private alleles shared with every other CAHG population using ADZE **(18)**. Since ADZE is particularly designed to standardise datasets in which different groups are unevenly sampled, we ran our analyses for maximum standardized sample size values between 2-9. See also Schlebusch et al. **(19)** for an example of the use of this same method to evaluate private allele sharing within and between Khoe-San populations.

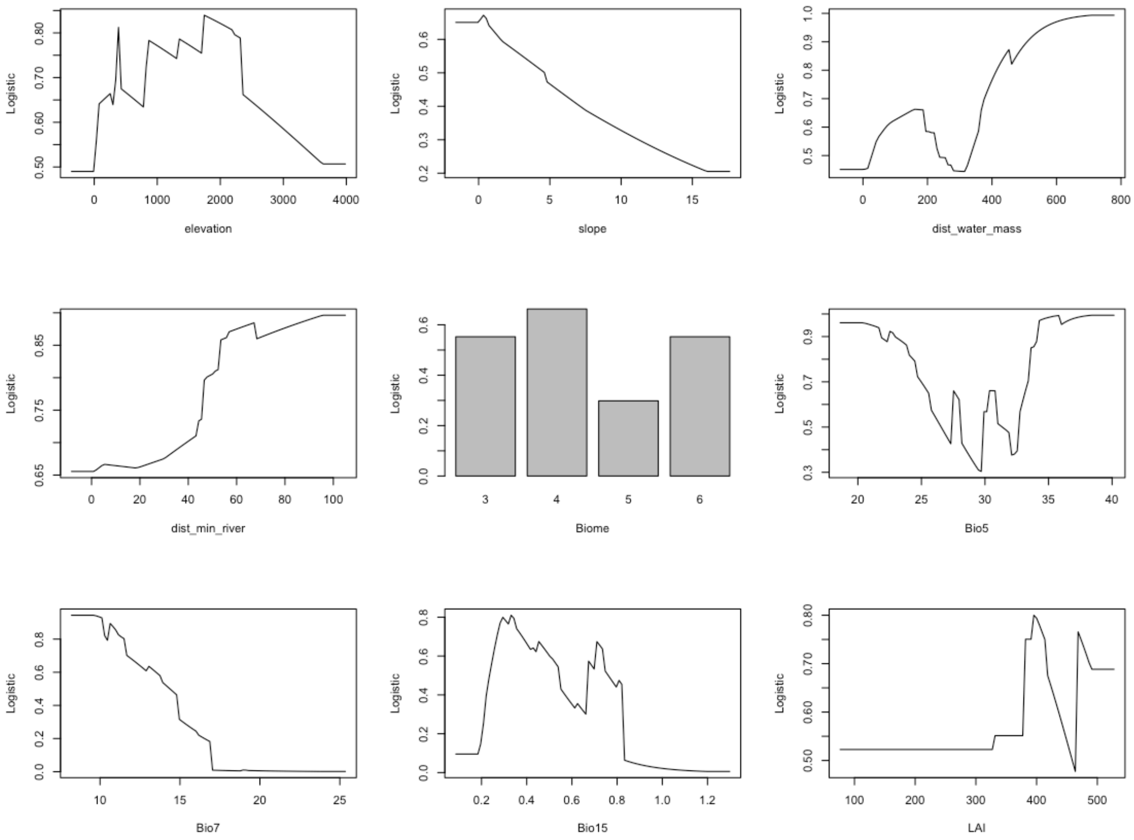


**Fig. S1.** Partial dependence plot of logistic output of MaxEnt model including environmental predictors only on the suitability of cells for CAHG presence.

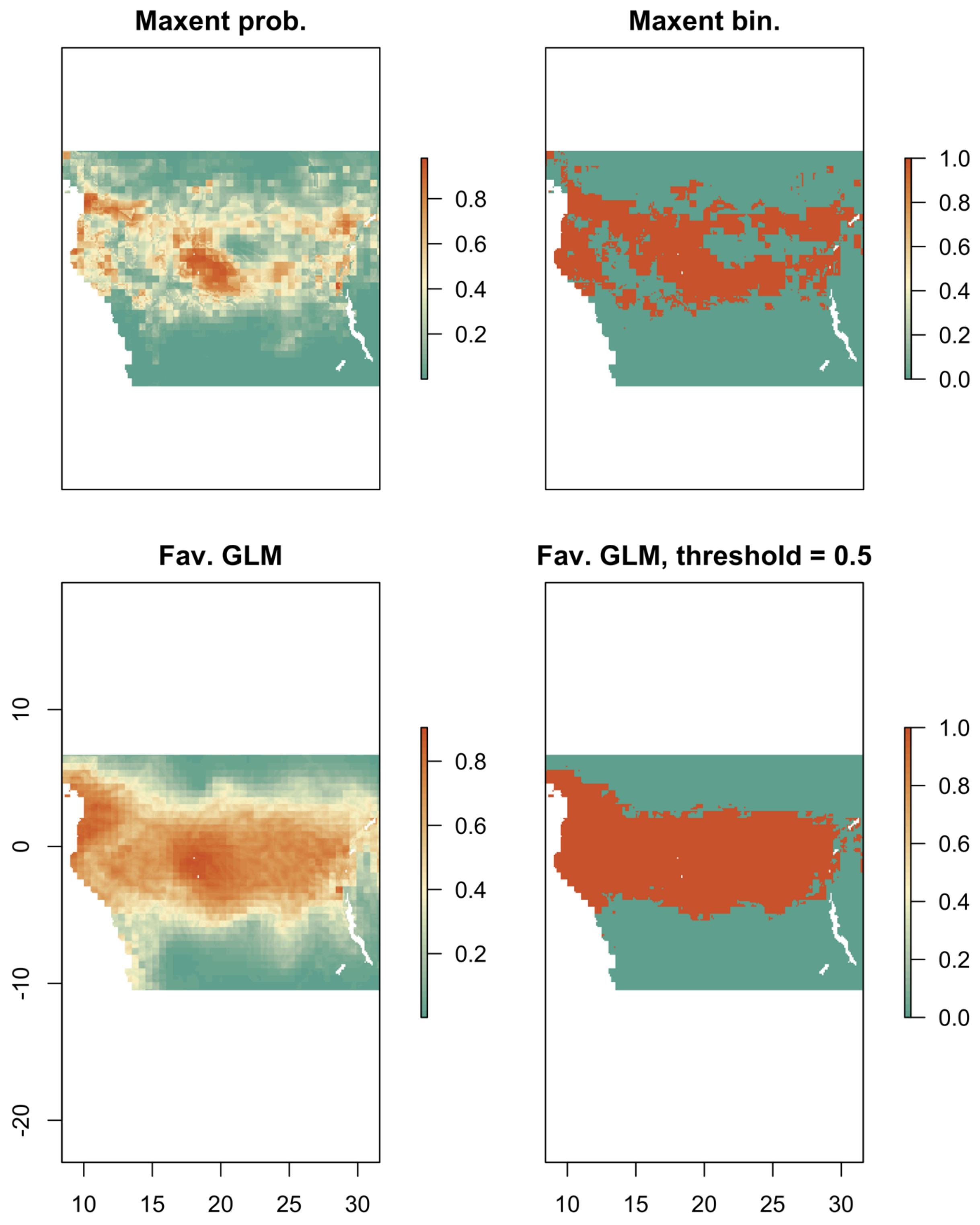


**Fig. S2.** Present suitability (top) and favourability (bottom) maps from our ENM. The right panels show on red predicted presences and in blue predicted absences according to the optimum threshold binarization for MaxEnt and a threshold of F=0.5 for the Favourability GLM.

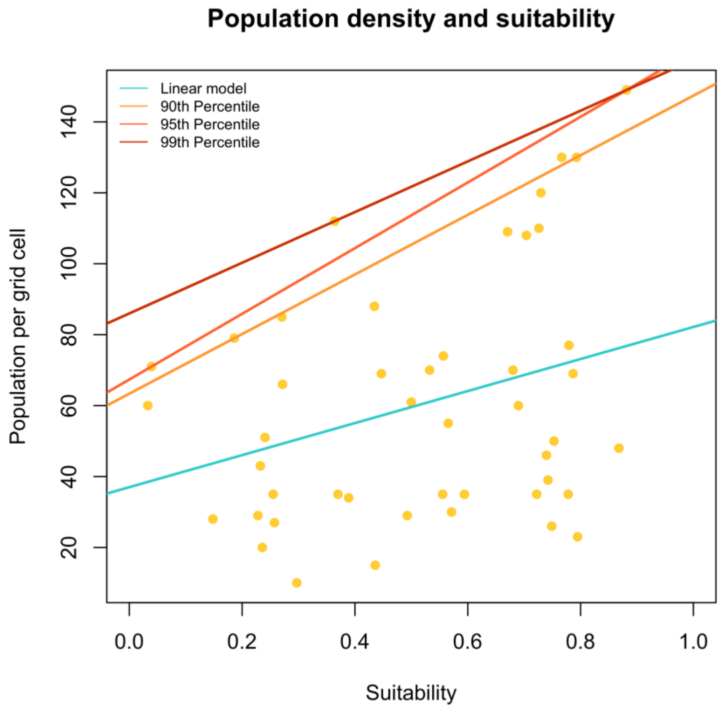


**Fig.S3.** Space defined by predicted environmental suitability (x-axis) and grid cell population size (y-axis). The yellow dots represent the N=50 grid cells containing camps for which the number of occupants was known (N=75). The red and orange lines fit the quantile regressions with the 90$^{th}$, 95$^{th}$ and 99$^{th}$ percentiles, representing the upper limit of potential population size. The blue line fits the linear regression between grid cell suitability and population density.

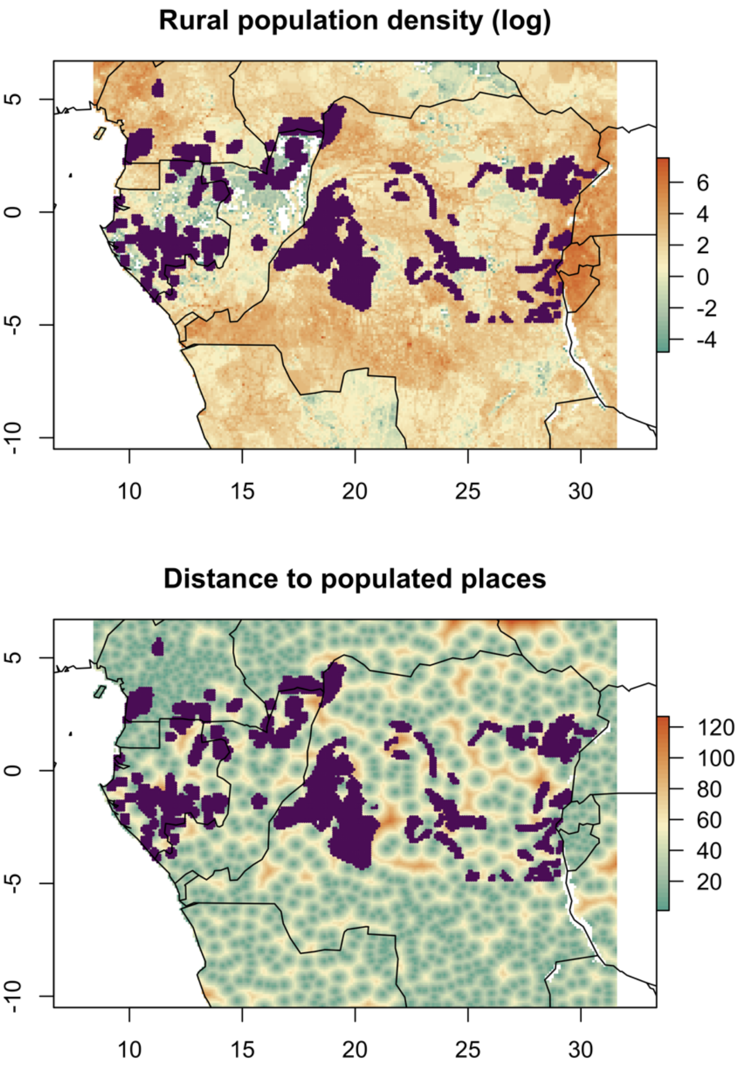


**Fig. S4.** Map of Rural population density (log people/km$^2$; top) and distance to populated places (bottom). Location of cells with CAHG presence is marked in purple.

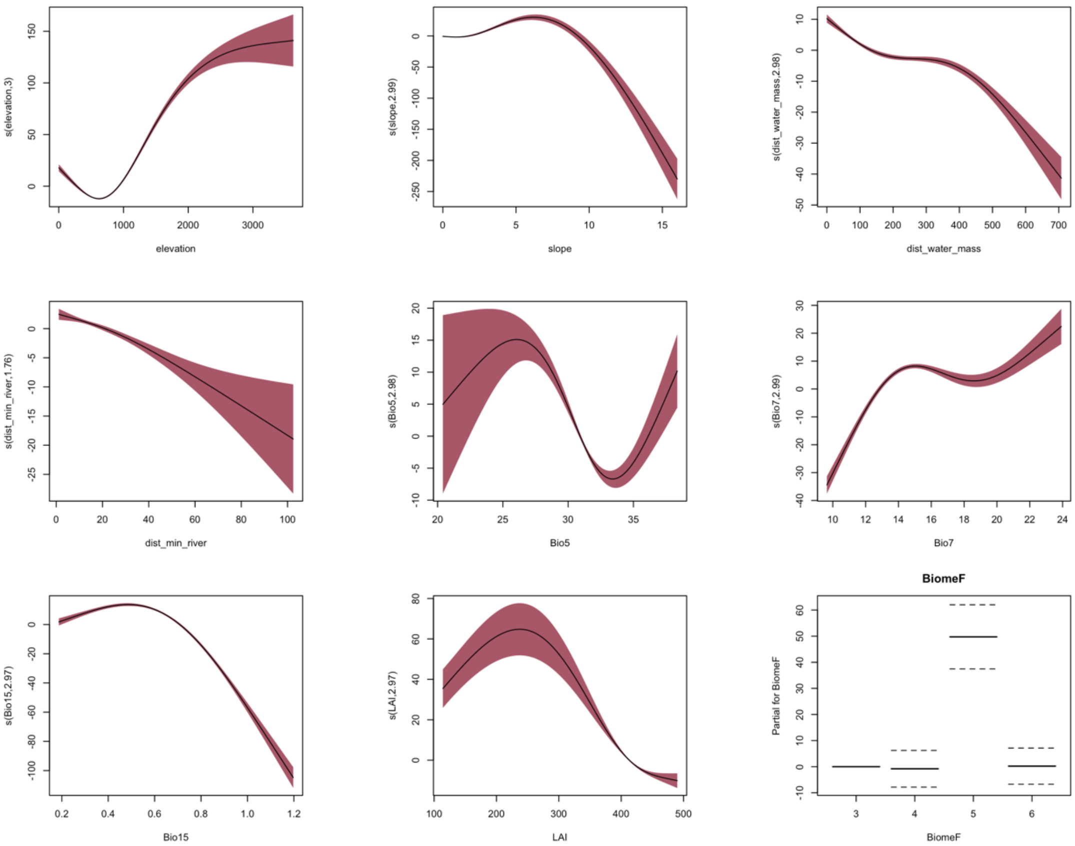


**Fig. S5.** Component smooth functions of GAM assessing the environmental predictors of rural population density. Panel labelled BiomeF shows term plot for “Biome”, also included as parametric component in the model.

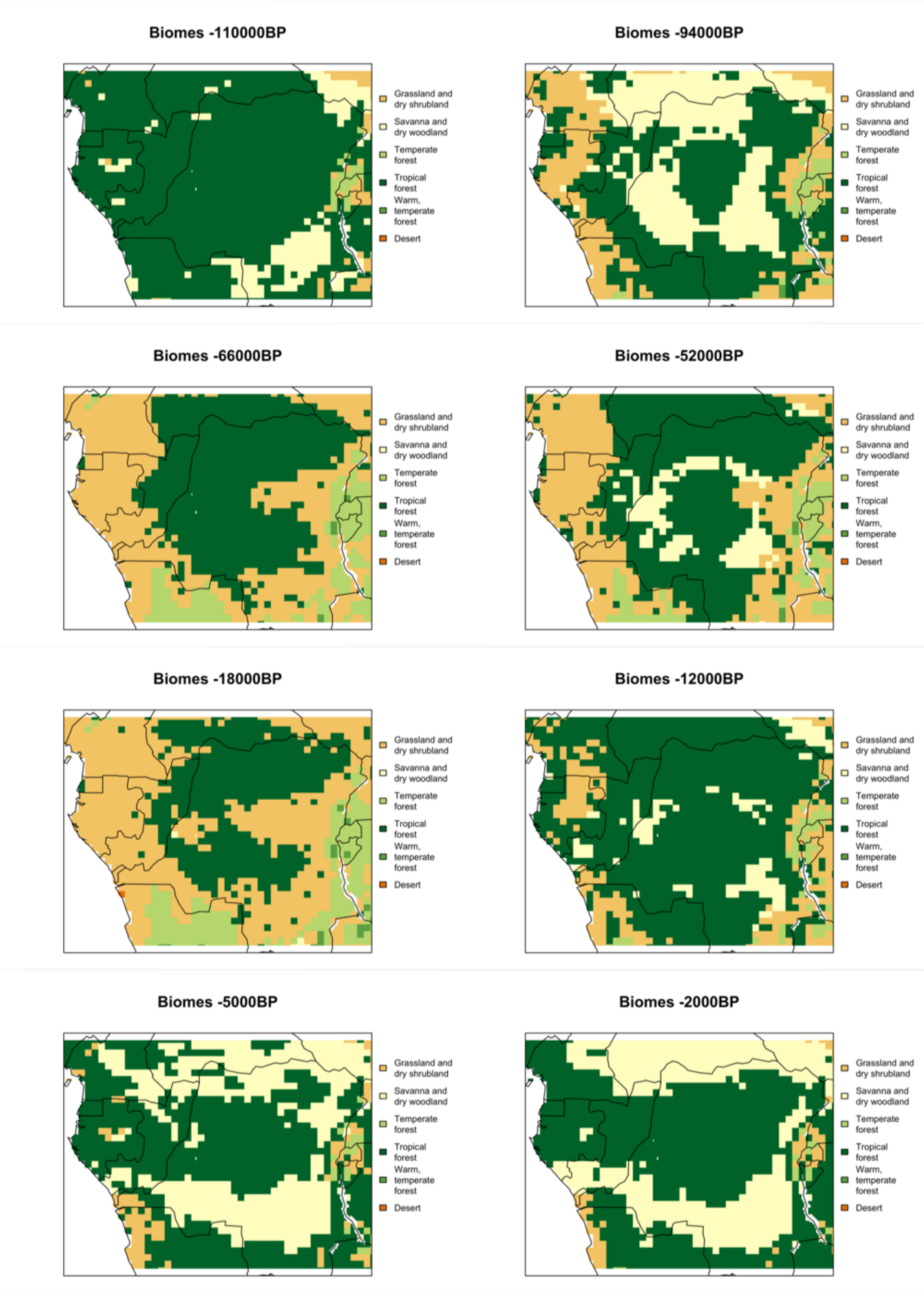


**Fig. S6.** Distribution of reconstructed biomes across the area of study according to Beyer et al. (20)

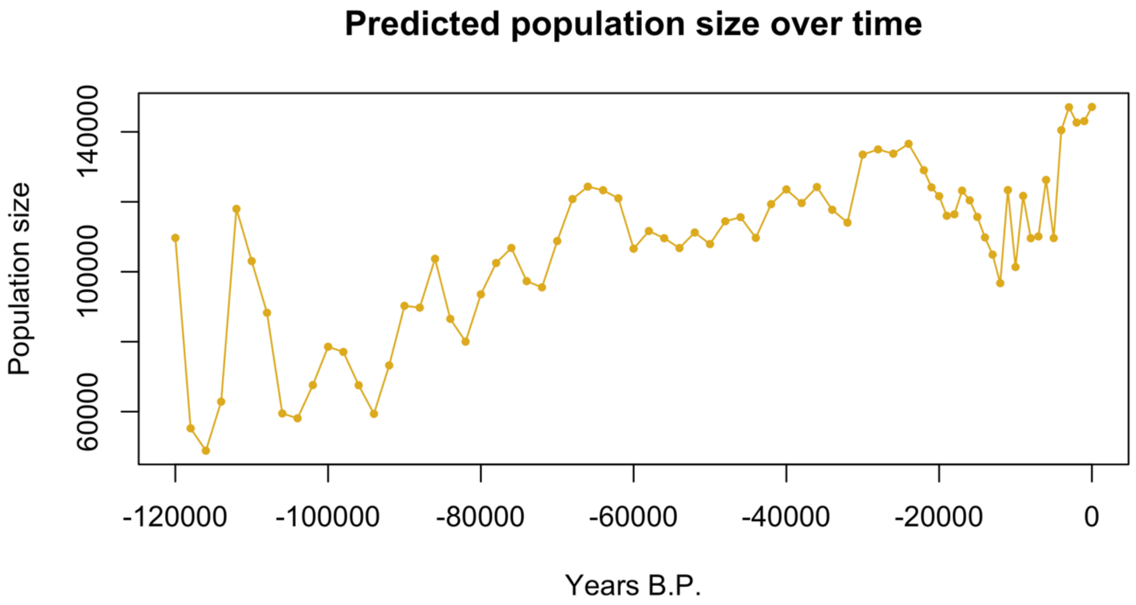


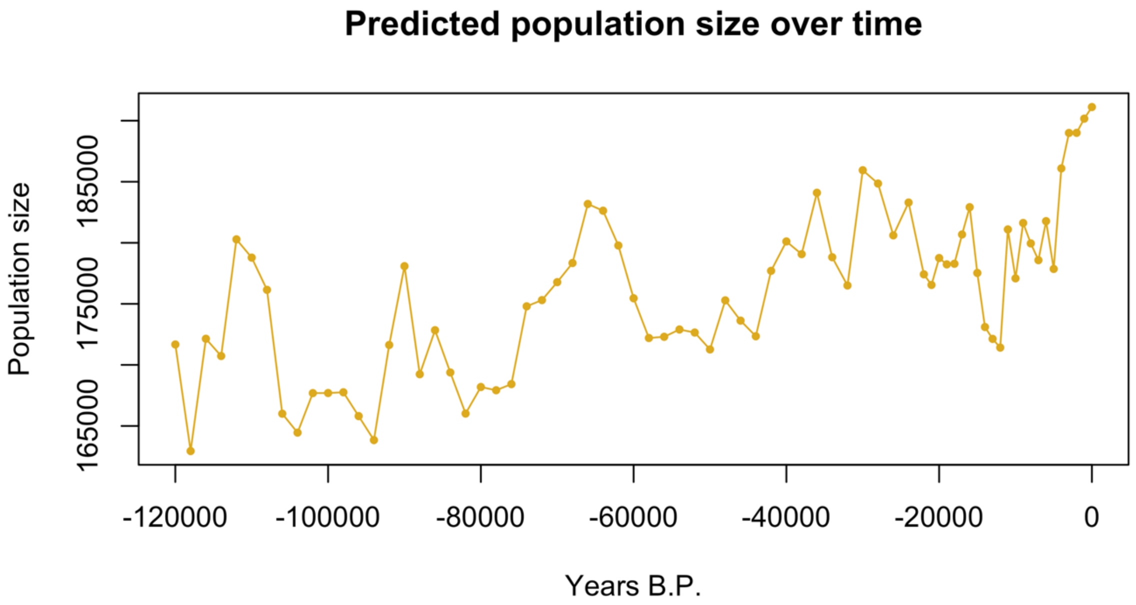


**Fig.S7.** Predicted population size over time using the favourability categories (top) and suitability regression (bottom).

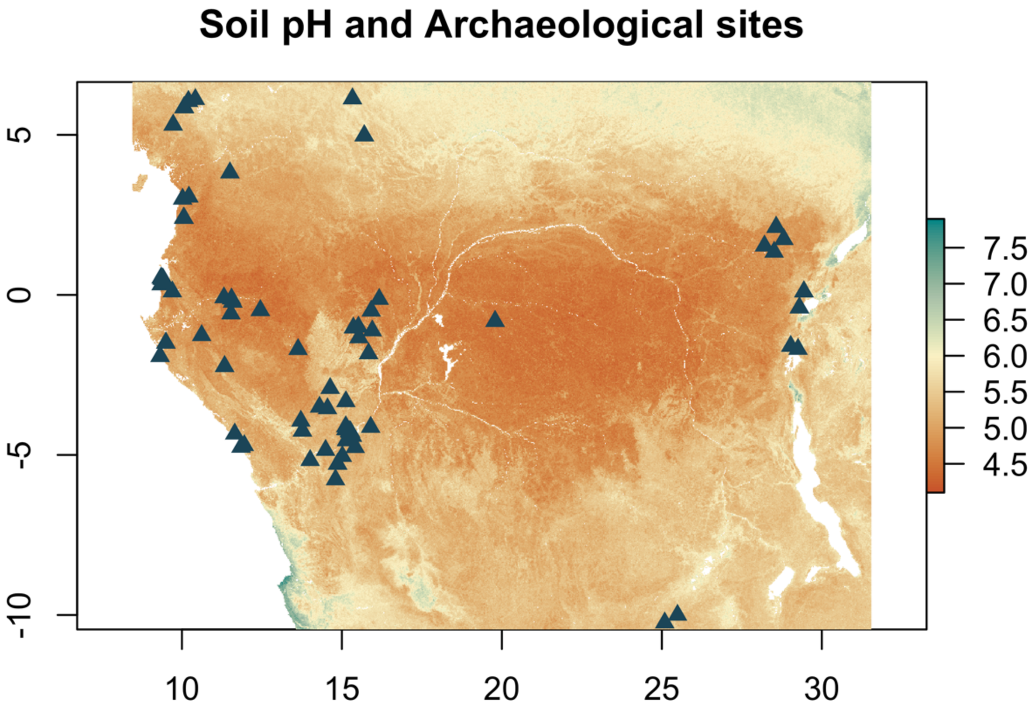


**Fig.S8.** Relationship between archaeological site location (blue triangles) and soil pH. pH data obtained from SoilGrids (21).

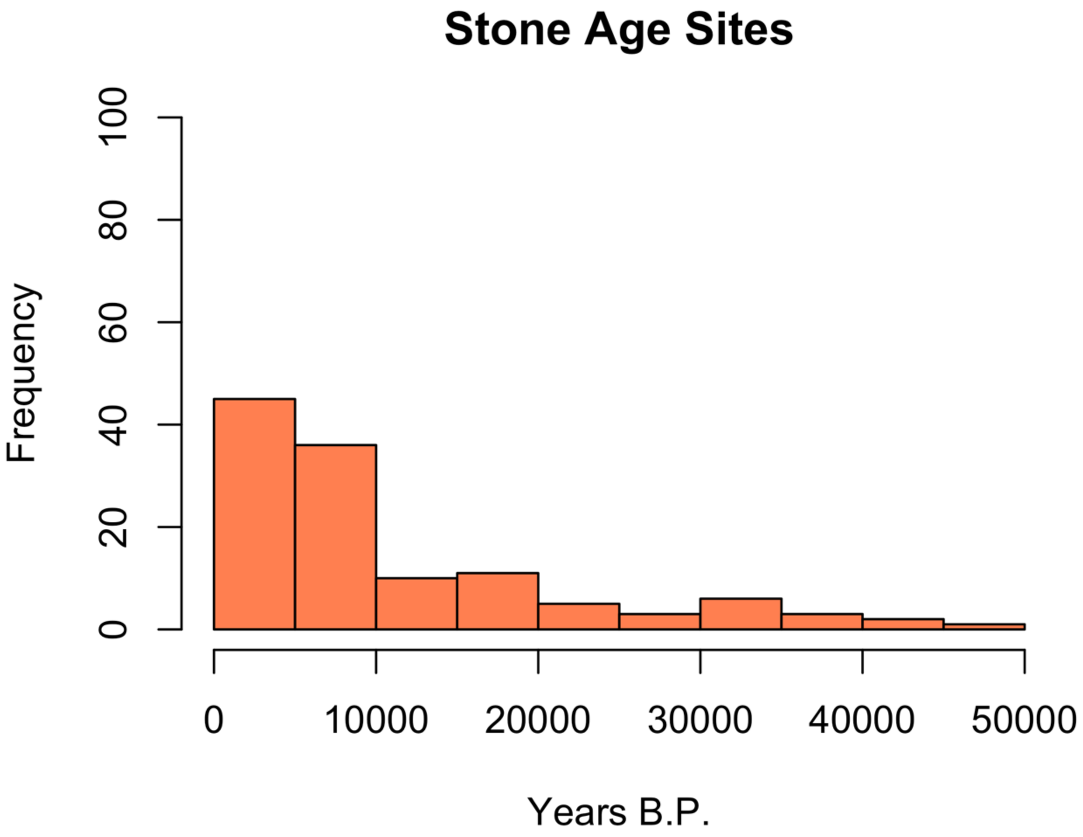


**Fig.S9.** Distribution of $^{14}C$ dates included in this study.

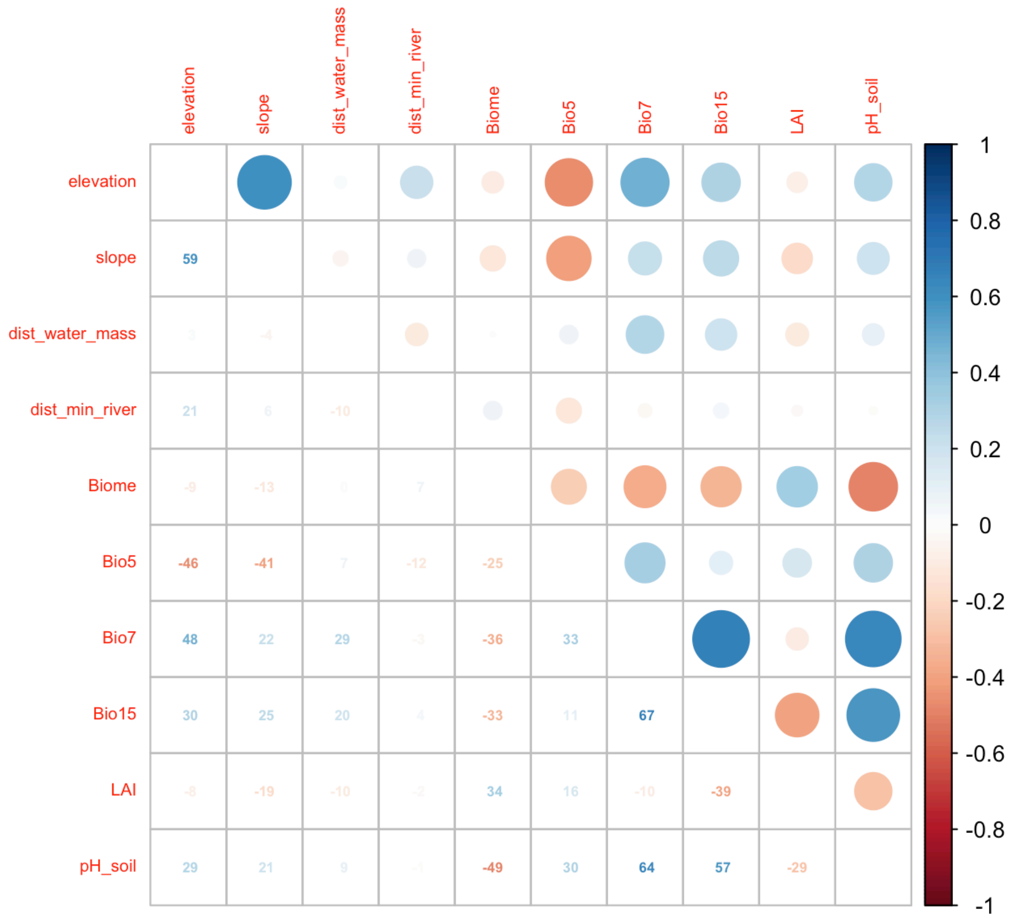


**Fig.S10**. Bivariate correlations between variables included in our ENM and soil pH. Data from soil pH was obtained from SoilGrids (21).

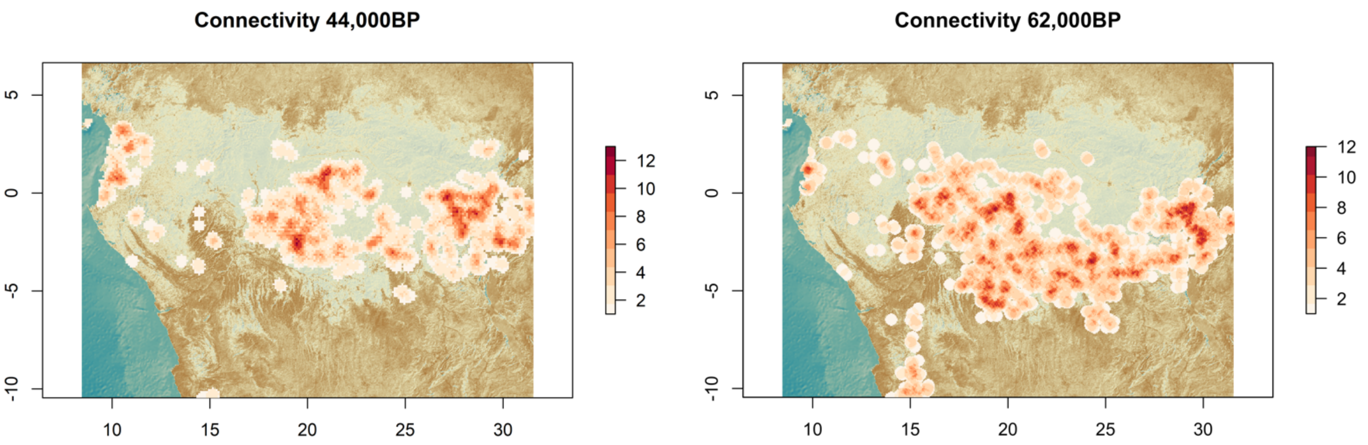


**Fig.S11.** Predicted connectivity from our MaxEnt model at the time period corresponding to the genetically-derived estimates of population divergence times between Eastern and Western CAHG by Fan et al. (22); left) as well as by Bergström et al ((23); right). The predicted number of camps at each time period was randomly distributed across the cells with predicted presences, and the number of other camps within a 7hr walk of each camp calculated. Darker shading indicates a greater number of camps within a 7hr walking distance of one another.

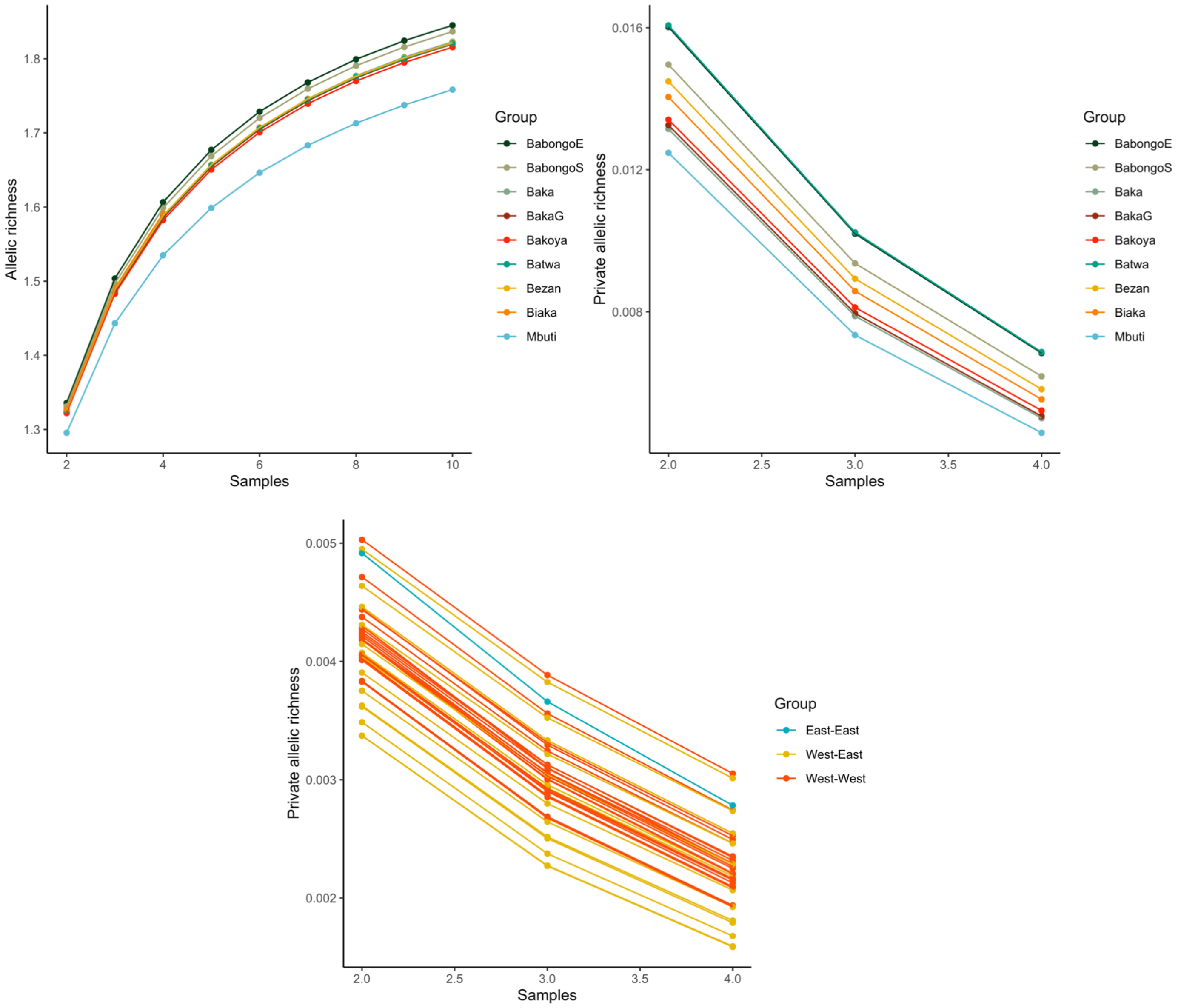


**Fig.S12.** Allele sharing (ADZE (18)). On the top left, allelic richness per CAHG group. On the top right, private allelic richness per CAHG group. At the bottom, private allelic richness (per variable site) of alleles shared by pairwise combinations of the 9 CAHG populations.

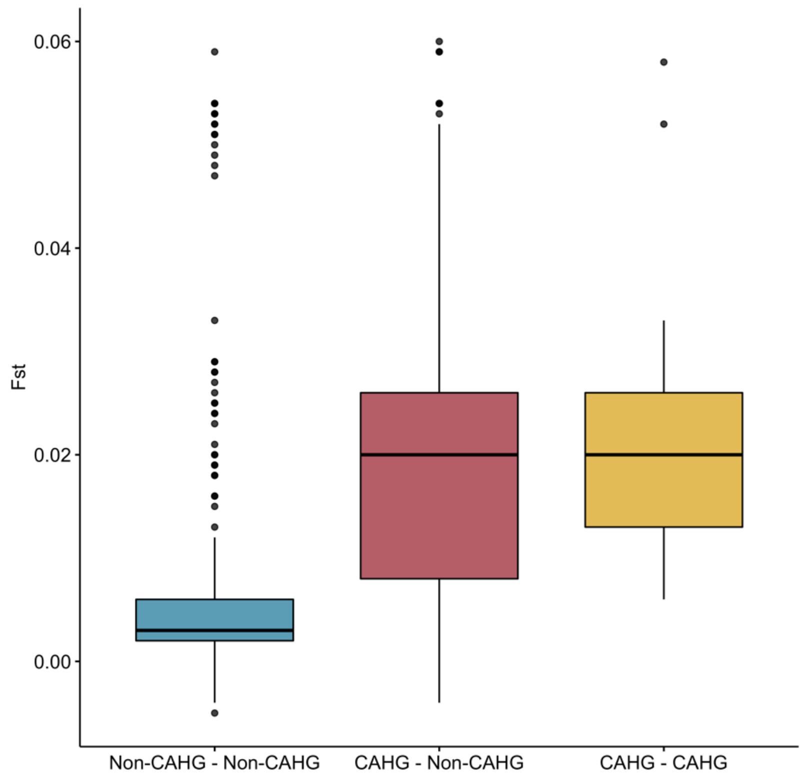


**Fig.S13.** Pairwise $F_{ST}$ values between the N=40 populations included in our study.

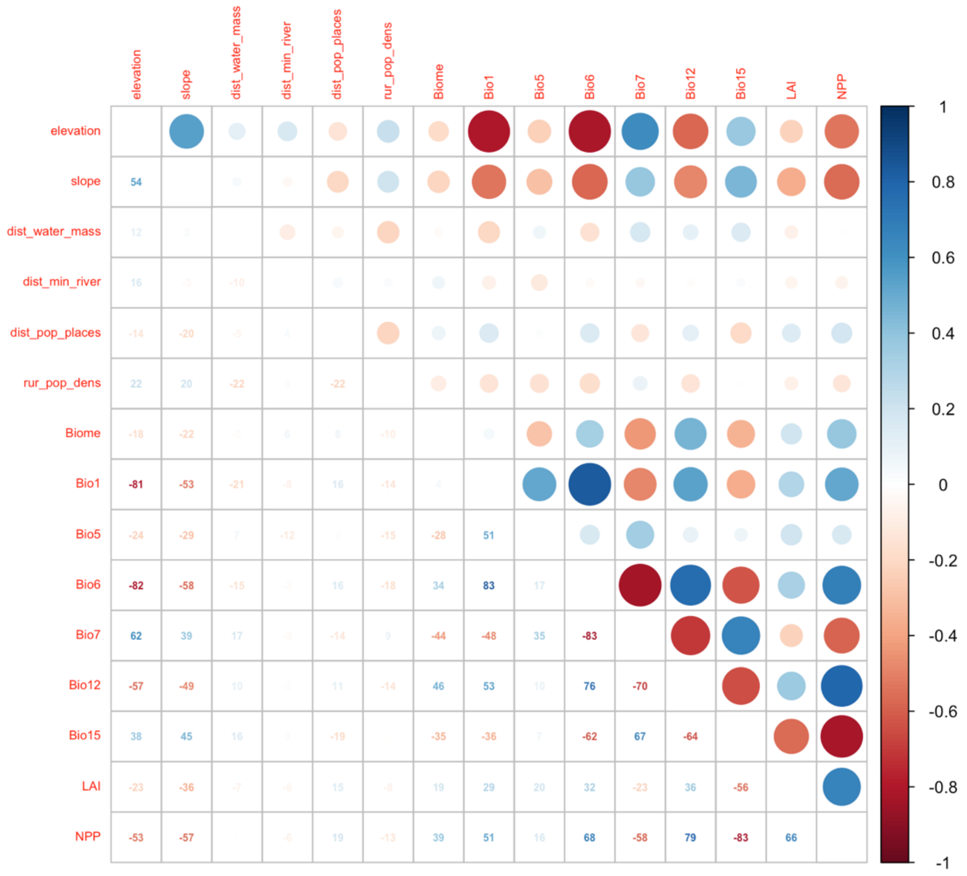


**Fig. S14.** Bivariate correlations between variables considered relevant for hunter-gatherer presence before using the *select_07* function.

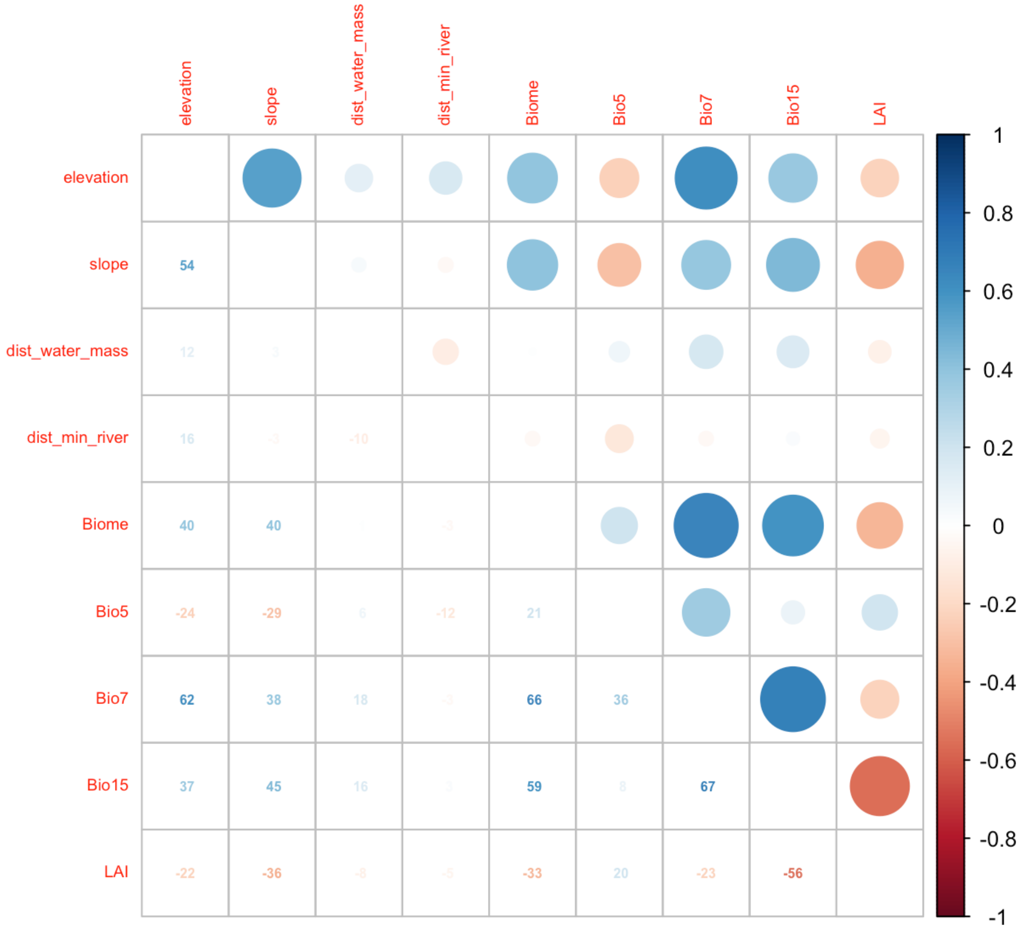


**Fig. S15.** Bivariate correlations between variables used for building our model selected based on AIC values with the function *select_07*

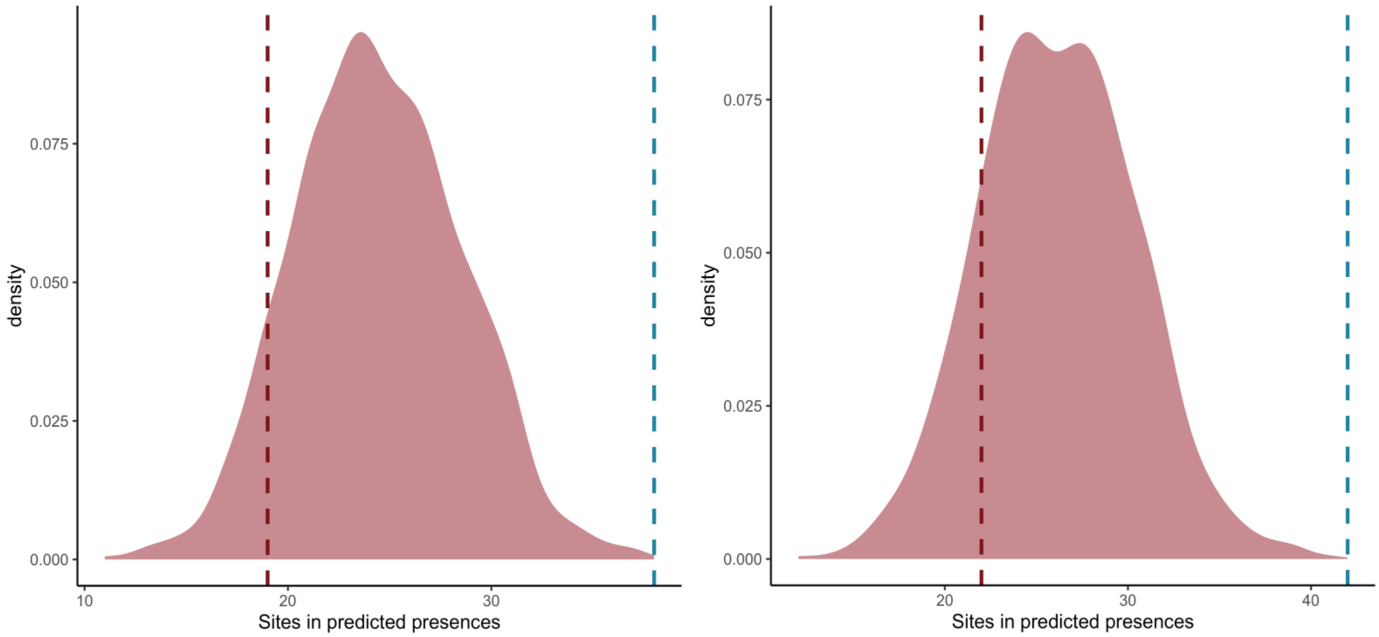


**Fig.S16.** MaxEnt predicted number of archaeological sites in suitable cells (blue line) in the dataset excluding sites from Gombe Point and Rivière Denis (left) and in the dataset excluding all sites with evidence of pottery use (right). Distribution shows number of sites in predicted presences across 1000 randomisations of site dates. Dashed pink line indicates expected cumulative number of sites in predicted presences when randomising their spatial location at each time period.

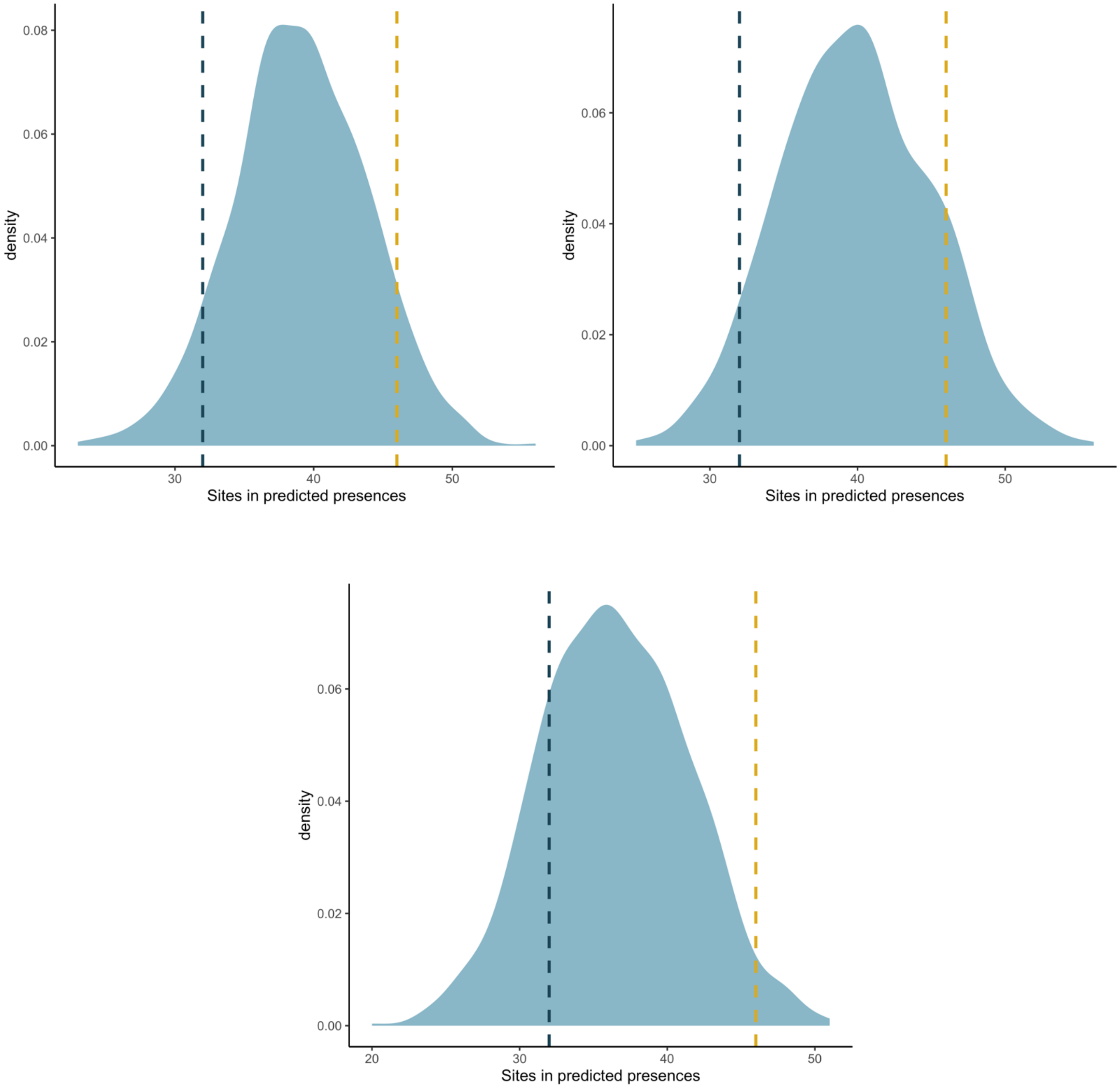


**Fig.S17.** From our favourability GLM predicted number of archaeological sites in favourable cells (yellow line) in the full dataset (top left), in the dataset excluding sites from Gombe Point and Rivière Denis (top right) and in the dataset excluding all sites with evidence of pottery use (bottom). Distribution shows number of sites in predicted presences across 1000 randomisations of site dates. Dashed pink line indicates expected cumulative number of sites in predicted presences when randomising their spatial location at each time period.

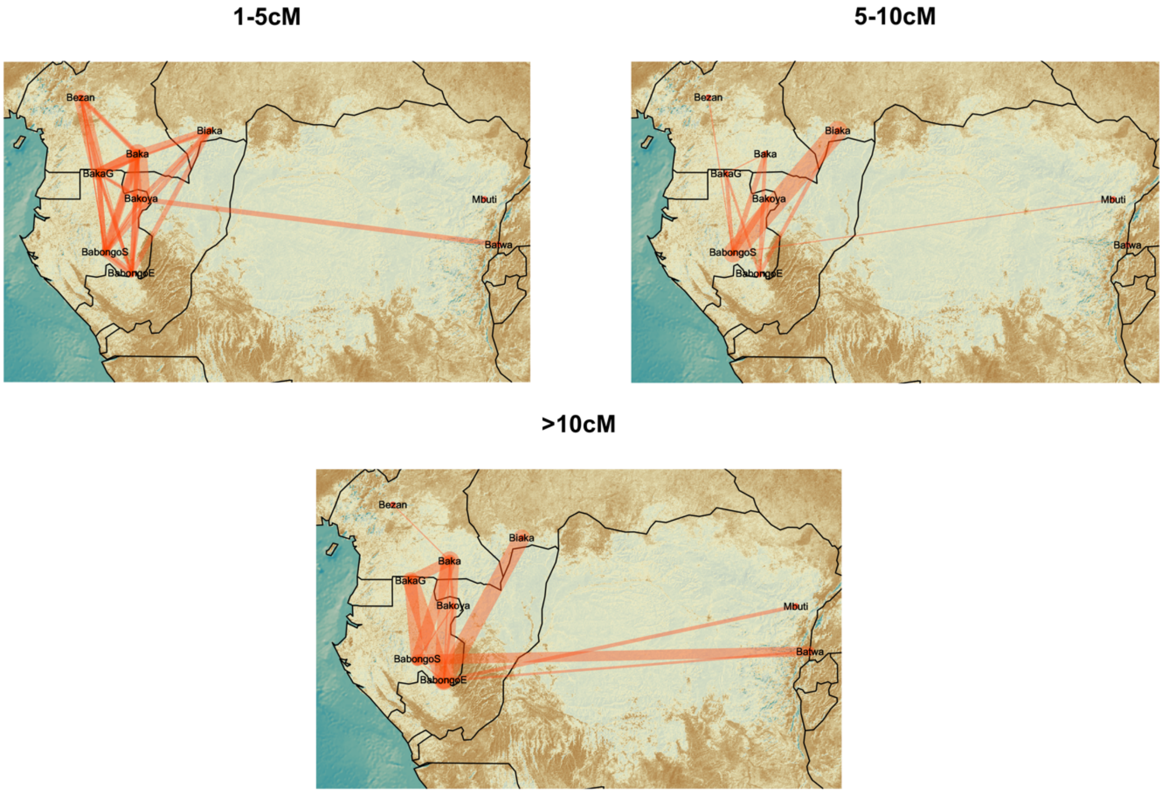


**Fig.S18.** Recent genetic connectivity between CAHG populations. Network visualizations of the average number of IBD segments shared per cross-population individual pairs including centromere and telomere regions with identified IBD blocks in the range of: 1–5 cM (top left), 5–10 cM (top right) and over 10 cM (bottom).

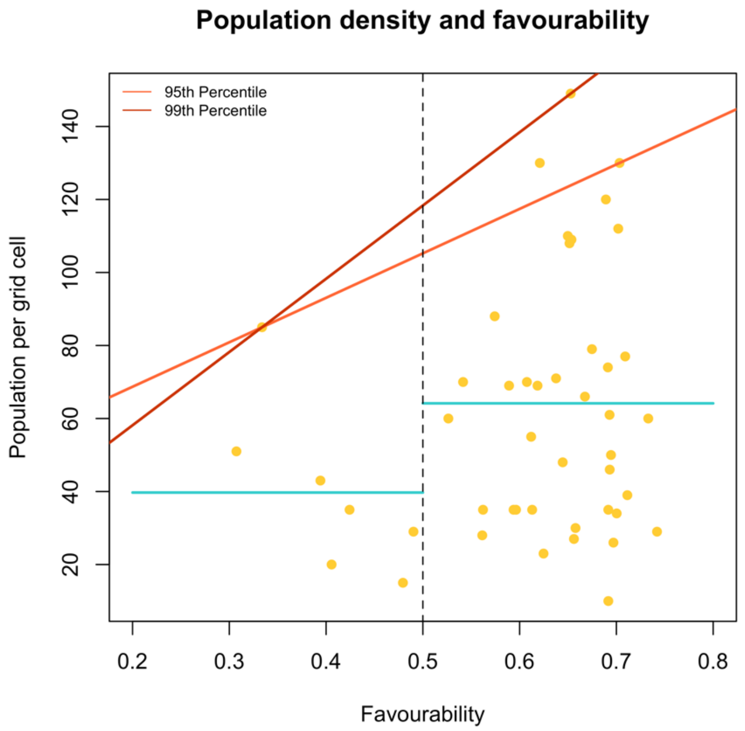


**Fig.S19.** Space defined by predicted environmental favourability (x-axis) and grid cell population size (y-axis). The yellow dots represent the N=50 grid cells containing camps for which the number of occupants was known (N=75). The red and orange lines fit the quantile regressions with the 95$^{th}$ and 99$^{th}$ percentiles, representing the upper limit of potential population size. The blue lines indicate the mean grid cell population size for the favourability intervals 0.2 >= F > 0.5 and 0.5>= F > 0.8.

**Table S1.** Estimates of separation times from genomic studies including CAHG populations. Studies marked with an asterisk do not rely on whole-genome data.

| Demographic event | Date | Reference | Method |
|---|---|---|---|
| Separation between CAHG groups and San | 68,000BP [52,000-100,000]<br><br><br><br><br>87,000BP [59,000-100,000]<br>87,000BP [59,000-120,000]<br>87,000BP [53,000-100,000]<br>68,000BP [53,000-100,000]<br>70,000BP [45,000-100,000]<br>70,000BP [55,000-100,000]<br>60,000BP [53,000-100,000] | Fan et al. 2019 | MSMC (Midpoint of cross-coalescence rate); Populations included: CAHG: Biaka, Baka; San: Khomani San<br>Mbuti – Khomani San<br>Bedzan – Khomani San<br>Bakola – Khomani San<br>Biaka, Mbuti – Juhoansi<br>Baka – Juhoansi<br>Bakola – Juhoansi<br>Bedzan – Juhoansi |
| | 110,000 BP [55,000-120,000] | Bergström et al. 2020 | MSMC2 (Midpoint of cross-coalescence) Populations: CAHG: Biaka, Mbuti. |
| | 105,000BP [70,000 -190,000] | Wang et al. 2020 | MSMC-IM (Point where 50% of ancestry has merged) |
| Separation between CAHG groups and Central African Bantu | 56,000BP [26,000-84,000]<br>44,000BP [26,000-70,000] | Mallick et al. 2016 | MSMC; Populations included: CAHG = Mbuti, Bantu = Luhya and Bantu = Bantu Kenya (respectively) |
| | 135,140BP [57,594-259,243],<br>97,585BP [30,323-134,807],<br>116,638BP [37,227-141,043] | López et al 2018* | fastsimcoal on exome data (Model parameters estimated by maximum likelihood and 95% confidence intervals – the 3 different estimates are from 3 different demographic models); Populations included: CAHG = Batwa and Mbuti; Bantu = Bakiga, Bapunu, Nzebi |
| | 215,000BP [204,000-219,000] | Schlebusch et al. 2020 | TT-Method (pairwise sampling under split model) |
| Separation between Eastern and Western CAHG groups | 44,000BP [31,000-50,000] | Fan et al. 2019 | MSMC; Populations included: West = Bedzan, Biaka, Baka and Bakola; East = Mbuti |
| | 62,000BP [45,000-80,000] | Bergström et al. 2020 | MSMC2; Populations included: West = Biaka, East = Mbuti |
| | 38,000 [27,000-44,000] | Mallick et al. 2016 | MSMC; Populations included: West = Biaka, East = Mbuti |
| | 19,749BP [10,499-27,454],<br>18,270BP [7,156-29,889],<br>18,154BP [3,693-36,273] | López et al 2018* | fastsimcoal on exome data; Populations included: East = Batwa, West = Baka. |
| Separation between Western CAHG populations | 12,000BP [12,000-18,000] | Fan et al. 2019 | MSMC (Midpoint of cross-coalescence) Populations considered: Biaka, Baka, Bedzan, Bakola |

**Table S2.** Model performance of MaxEnt and Favourability ENMs.

| Model | Sensitivity | Specificity | TSS | AUC | Kappa |
|---|---|---|---|---|---|
| **MaxEnt Environment only** | **0.81** | **0.74** | **0.55** | **0.87** | **0.33** |
| MaxEnt Environment only (no minor rivers) | 0.78 | 0.78 | 0.56 | 0.87 | 0.37 |
| MaxEnt Environment + distance from populated places | 0.70 | 0.81 | 0.51 | 0.85 | 0.36 |
| MaxEnt Environment + rural population density | 0.85 | 0.68 | 0.51 | 0.85 | 0.28 |
| **Favourability Environment only** | **0.83** | **0.66** | **0.49** | **0.80** | **0.25** |
| Favourability Environment only (no minor rivers) | 0.84 | 0.65 | 0.49 | 0.80 | 0.25 |
| Favourability Environment + distance from populated places | 0.83 | 0.66 | 0.48 | 0.80 | 0.25 |
| Favourability Environment + rural population density | 0.84 | 0.66 | 0.50 | 0.80 | 0.26 |

**Table S3.** Variable importance from MaxEnt model ordered from most to least important

| | **Permutation importance** |
|---|---|
| Bio7 | 0.76 |
| Bio15 | 0.186 |
| Bio5 | 0.108 |
| Distance to water masses | 0.024 |
| LAI | 0.018 |
| Elevation | 0.01 |
| Minimum distance to rivers | 0.005 |
| Biome | 0.004 |
| Slope | 0.002 |

**Table S4.** Coefficients from quantile regressions of suitability against grid cell population density in the 50 grid cells for which population size data were available.

| Percentile | Value | t | Pr(>\|t\|) | $R^2$ |
|---|---|---|---|---|
| $99^{th}$ | 74.86 | 16070510 | <0.001 | 0.32 |
| $95^{th}$ | 94.35 | 3.02 | 0.04 | 0.24 |
| $90^{th}$ | 75.07 | 3.96 | <0.001 | 0.16 |

**Table S5.** Coefficients from GAM predicting rural population density with the same predictors used for the ENMs. Reference Biome = Biome3.

| **Parametric coefficients** | | | | |
|---|---|---|---|---|
| | **Estimate** | **Std. Error** | **t value** | **Pr(>\|t\|)** |
| (Intercept) | 20.780 | 3.326 | 6.248 | 0.000 |
| Biome4 | -0.804 | 3.512 | -0.229 | 0.819 |
| Biome5 | 49.738 | 6.144 | 8.095 | 0.000 |
| Biome6 | 0.211 | 3.460 | 0.061 | 0.951 |

| **Smooth terms** | | | | |
|---|---|---|---|---|
| | **edf** | **Ref.df** | **F** | **P-value** |
| s(elevation) | 2.997 | 3.000 | 748.020 | 0.000 |
| s(slope) | 2.994 | 3.000 | 116.870 | 0.000 |
| s(dist_water_mass) | 2.980 | 3.000 | 119.080 | 0.000 |
| s(dist_min_river) | 1.764 | 2.153 | 31.770 | 0.000 |
| s(Bio5) | 2.977 | 3.000 | 66.440 | 0.000 |
| s(Bio7) | 2.993 | 3.000 | 192.960 | 0.000 |
| s(Bio15) | 2.965 | 2.999 | 520.280 | 0.000 |
| s(LAI) | 2.969 | 2.999 | 62.550 | 0.000 |

**Table S6.** Details on *Homo sapiens* skeletal remains found in the region of interest. Asterisk indicates that the dating of the remains has been contested following its original publication.

| Site name | Date(s) | Details | Source |
|---|---|---|---|
| Ishango 11 | 22,000BP and 20,000BP | 138 specimens representing at least 12 different individuals of multiple age groups. | Twiesselmann, 1958; Crevecoeur et al. 2016 |
| Matangai Turu NW | 810BP | Single skeleton associated with Late Stone Age lithics. | Mercader et al. 2001 |
| Matupi Cave* | 16,740 BP and 12,050 BP | In the oldest layer: A lower deciduous canine with a resorbed root. In the youngest layer: Postcranial remains, indicator of a burial site. Bone density much younger than surroundings – potentially indicating that the remains are younger than that. | Van Neer, 1989 |
| Shum Laka | 6,985BP, 7,090BP, 2,940BP and 2,970BP | Four children. Ancient DNA extracted from the remains confirms that these individuals are most closely related to contemporary CAHGs than to any other human population. | Lipson et al. 2020 |
| Ntadi Yomba | 7,000BP | 9 bones, four of which belong to children. | Van Neer and Lanfranchi, 1985 |
| Mbi Crater | 7,790 BP | Single skeleton. | De Maret et al. 1977 |

**Table S7.** Populations included in our genetic analyses. Asterisks denote CAHG populations.

| Population | Number of individuals |
|---|---|
| Ahizi | 20 |
| Akele | 41 |
| Babongo East* | 40 |
| Babongo South* | 25 |
| Badwee | 39 |
| Baka Cameroon* | 129 |
| Baka Gabon* | 30 |
| Bakiga | 61 |
| Bakota | 50 |
| Bakoya* | 25 |
| Bapunu | 49 |
| Bariba | 20 |
| Bateke | 44 |
| Batwa* | 96 |
| Bekwil | 5 |
| Benga | 46 |
| Bezan* | 28 |
| Biaka* | 18 |
| Duma | 43 |
| Eshira | 41 |
| Eviya | 31 |
| Fang | 68 |
| Fon | 12 |
| Galoa | 49 |
| Kimbundu | 17 |
| Kongo | 10 |
| Luhya | 74 |
| Makina | 41 |
| Mandinka | 86 |
| Mbuti* | 15 |
| Ndumu | 37 |
| Nzebi | 60 |

| | |
|---|---|
| Nzime | 51 |
| Obamba | 46 |
| Okande | 8 |
| Orungu | 19 |
| Ovimbundu | 15 |
| Shake | 47 |
| Sotho | 83 |
| Tsogo | 60 |
| Yacouba | 17 |
| Yoruba | 19 |
| YorubaN | 100 |

**Table S8.** Average length (in cM) of shared IBD segments between- and within-CAHG populations.

| | BabongoE | BabongoS | Baka | BakaG | Bakoya | Batwa | Bezan | Biaka | Mbuti |
|---|---|---|---|---|---|---|---|---|---|
| **BabongoE** | 5.97 | | | | | | | | |
| **BabongoS** | 2.79 | 5.29 | | | | | | | |
| **Baka** | 2.16 | 2.22 | 4.25 | | | | | | |
| **BakaG** | 2.16 | 2.17 | 4.32 | 5.77 | | | | | |
| **Bakoya** | 2.15 | 2.18 | 2.39 | 2.38 | 4.64 | | | | |
| **Batwa** | 2.05 | 2.06 | 2.06 | 2.05 | 2.09 | 5.90 | | | |
| **Bezan** | 2.12 | 2.15 | 2.41 | 2.41 | 2.41 | 2.06 | 6.34 | | |
| **Biaka** | 2.15 | 2.21 | 2.87 | 2.85 | 2.41 | 2.03 | 2.42 | 4.27 | |
| **Mbuti** | 2.08 | 2.08 | 2.03 | 2.07 | 2.07 | 2.16 | 2.13 | 1.94 | 3.59 |

**Table S9.** Variables considered relevant for the presence of hunter-gatherers and inputted to the *select_07* function.

| Variable | Unit | Source |
|---|---|---|
| **Bioclimatic variables** | | |
| BIO1: Annual mean temperature | °C | Beyer et al. 2020 |
| BIO5: Minimum annual temperature | °C | Beyer et al. 2020 |
| BIO6: Maximum annual temperature | °C | Beyer et al. 2020 |
| BIO7: Temperature annual range | °C | Beyer et al. 2020 |
| BIO12: Annual precipitation | mm $year^{-1}$ | Beyer et al. 2020 |
| BIO15: Precipitation seasonality | — | Beyer et al. 2020 |
| **Vegetation variables** | | |
| Net primary productivity | gC $m^{-2}$ $year^{-1}$ | Beyer et al. 2020 |
| Leaf area index | gC $m^{-2}$ | Beyer et al. 2020 |
| Biome | categorial | Beyer et al. 2020 |
| **Topographical variables** | | |
| Elevation | m | GTOPO30; US Geological Survey, 1996 |
| Slope | — | GTOPO30; US Geological Survey, 1996 |
| **Hydrographical variables** | | |
| Distance to water masses (lakes and rivers with water flow accumulation > $10^6$ cells) | km | http://hydrosheds.cr.usgs.gov |
| Distance to minor rivers (with water flow accumulation of $10^4$ - $10^6$ cells | km | http://hydrosheds.cr.usgs.gov |
| **Variables related to farmer populations** | | |
| Rural population density (excluding areas < 2km away from urban areas) | people/$km^2$ | Schneider et al. 2009; 2010 |
| Distance to populated places | km | http://www.fao.org/geonetwork |

**Table S10.** Favourability GLM model coefficients. Reference Biome = Biome3 (Grassland and Dry Shrubland).

| | Estimate | Std. Error | z value | Pr(>\|z\|) |
|---|---|---|---|---|
| (Intercept) | 8.739 | 0.665 | 13.150 | 0.000 |
| Elevation | 0.000 | 0.000 | 2.087 | 0.037 |
| Slope | -0.059 | 0.024 | -2.482 | 0.013 |
| Distance to water masses | 0.000 | 0.000 | 0.473 | 0.636 |
| Minimum distance to rivers | 0.007 | 0.001 | 5.088 | 0.000 |
| Biome4 (Savanna and dry woodland) | 1.216 | 0.186 | 6.523 | 0.000 |
| Biome5 (Temperate forest) | 0.947 | 0.285 | 3.325 | 0.001 |
| Biome6 (Tropical forest) | 0.911 | 0.184 | 4.947 | 0.000 |
| Bio5 | -0.050 | 0.022 | -2.248 | 0.025 |
| Bio7 | -0.561 | 0.018 | -30.584 | 0.000 |
| Bio15 | -1.390 | 0.139 | -10.013 | 0.000 |
| LAI | -0.005 | 0.001 | -5.421 | 0.000 |

**Movie S1.**
Predicted suitability ranges from MaxEnt model at each time snap.

**Dataset S1. (separate file)**
Hunter-gatherer camps included in our study.

**Dataset S2. (separate file)**
Hunter-gatherer archaeological sites included in our study.

**Dataset S3. (separate file)**
Initial list of all $^{14}C$ dates from our area of interest from the Late Pleistocene to the present.

**Dataset S4. (separate file)**
Final list of archaeological sites included in our study after merging multiple dates within the same grid cell and 1000- or 2000-year time interval.

## SI References


1. J. Olivero, *et al.*, Distribution and Numbers of Pygmies in Central African Forests. *PLOS ONE* **11**, e0144499 (2016).

2. C. Finlayson, *The improbable primate: how water shaped human evolution* (Oxford University Press, 2014).

3. R. D. Sagarin, S. D. Gaines, Geographical abundance distributions of coastal invertebrates: using one-dimensional ranges to test biogeographic hypotheses. *Journal of Biogeography* **29**, 985–997 (2002).

4. R. Real, A. M. Barbosa, J. M. Vargas, Obtaining environmental favourability functions from logistic regression. *Environmental and Ecological Statistics* **13**, 237–245 (2006).

5. P. Acevedo, R. Real, Favourability: concept, distinctive characteristics and potential usefulness. *Naturwissenschaften* **99**, 515–522 (2012).

6. A.-R. Muñoz, A. Jiménez-Valverde, A. L. Márquez, M. Moleón, R. Real, Environmental favourability as a cost-efficient tool to estimate carrying capacity. *Diversity Distrib.* **21**, 1388–1400 (2015).

7. R. Koenker, J. A. Machado, Goodness of fit and related inference processes for quantile regression. *Journal of the american statistical association* **94**, 1296–1310 (1999).

8. T. Clay, R. Phillips, A. Manica, H. Jackson, M. Brooke, Escaping the oligotrophic gyre? The year-round movements, foraging behaviour and habitat preferences of Murphy's petrels. *Mar. Ecol. Prog. Ser.* **579**, 139–155 (2017).

9. D. Seidensticker, *et al.*, Population collapse in Congo rainforest from 400 CE urges reassessment of the Bantu Expansion. *Science Advances* **7**, eabd8352 (2021).

10. Y. Garcin, *et al.*, Early anthropogenic impact on Western Central African rainforests 2,600 y ago. *Proc Natl Acad Sci USA* **115**, 3261–3266 (2018).

11. R. Oslisly, *et al.*, Climatic and cultural changes in the west Congo Basin forests over the past 5000 years. *Philosophical Transactions of the Royal Society B: Biological Sciences* **368**, 20120304 (2013).

12. D. Cahen, J. Moeyersons, Subsurface movements of stone artefacts and their implications for the prehistory of Central Africa. *Nature* **266**, 812–815 (1977).

13. M. Budja, Ceramics among Eurasian hunter-gatherers: 32 000 years of ceramic technology use and the perception of containment. *Doc. praeh.* **43**, 61–86 (2016).

14. K. D. Lupo, *et al.*, Hunter-gatherers on the basin's edge: a preliminary look at Holocene human occupation of Nangara-Komba Shelter, Central African Republic. *Azania: Archaeological Research in Africa* **56**, 4–33 (2021).

15. B. Clist, *Gabon, 100 000 ans d'histoire* (Centre culturel français Saint-Exupéry : Sépia, 1995).

16. P. Jordan, *et al.*, Modelling the diffusion of pottery technologies across Afro-Eurasia: emerging insights and future research. *Antiquity* **90**, 590–603 (2016).

17. A. Testart, *et al.*, The Significance of Food Storage Among Hunter-Gatherers: Residence Patterns, Population Densities, and Social Inequalities [and Comments and Reply]. *Current Anthropology* **23**, 523–537 (1982).

18. Z. A. Szpiech, M. Jakobsson, N. A. Rosenberg, ADZE: a rarefaction approach for counting alleles private to combinations of populations. *Bioinformatics* **24**, 2498–2504 (2008).

19. C. M. Schlebusch, *et al.*, Khoe-San Genomes Reveal Unique Variation and Confirm the Deepest Population Divergence in Homo sapiens. *Molecular Biology and Evolution* **37**, 2944–2954 (2020).

20. R. M. Beyer, M. Krapp, A. Manica, High-resolution terrestrial climate, bioclimate and vegetation for the last 120,000 years. *Scientific Data* **7**, 1–9 (2020).

21. L. M. de Sousa, *et al.*, "SoilGrids 2.0: producing quality-assessed soil information for theglobe" (Soils and the natural environment, 2020) https:/doi.org/10.5194/soil-2020-65 (November 25, 2021).

22. S. Fan, *et al.*, African evolutionary history inferred from whole genome sequence data of 44 indigenous African populations. *Genome Biol* **20**, 82 (2019).

23. A. Bergström, *et al.*, Insights into human genetic variation and population history from 929 diverse genomes. *Science* **367** (2020).
